# Bioflocculant produced by *Bacillus* *velezensis* and its potential application in brewery wastewater treatment

**DOI:** 10.1038/s41598-022-15193-8

**Published:** 2022-06-29

**Authors:** Mayowa Agunbiade, Babatunde Oladipo, Adedeji Nelson Ademakinwa, Oluyemi Awolusi, Ibukun Modupe Adesiyan, Oluwaseun Oyekola, Olusola Ololade, Abidemi Ojo

**Affiliations:** 1 Applied Microbial and Biotechnology Institute, Cape Peninsula University of Technology, Cape Town, 7535 South Africa; 2 Department of Chemical Engineering, Cape Peninsula University of Technology, Cape Town, South Africa; 3 Department of Physical and Chemical Sciences, Elizade University, Ilara-Mokin, 002 Nigeria; 4 Institute for Water and Wastewater Technology, Durban University of Technology, Durban, 4000 South Africa; 5 Biological Sciences Department, Achievers University, Owo, Ondo State Nigeria; 6 Centre for Environmental Management, University of the Free State, Bloemfontein, 9301 South Africa; 7 Centre for Mineral Biogeochemistry, University of the Free State, Bloemfontein, 9301 South Africa

**Keywords:** Biotechnology, Environmental sciences

## Abstract

This study was designed to evaluate the potential of bioflocculant producing strains isolated from wastewater sludge. According to the Plackett–Burman design, the response surface revealed glucose, magnesium sulfate, and ammonium sulfate as critical media components of the nutritional source, whereas the central composite design affirmed an optimum concentration of the critical nutritional source as 16.0 g/l (glucose), 3.5 g/l magnesium sulfate heptahydrate (MgSO_4_.7H_2_O), and 1.6 g/l ammonium sulfate ( (NH_4_)_2_SO_4_), yielding an optimal flocculation activity of 96.8%. Fourier Transformer Infrared Spectroscopy (FTIR) analysis confirmed the presence of hydroxyl, carboxyl and methoxyl in the structure of the bioflocculant. Additionally, chemical analysis affirmed the presence of mainly a polysaccharide in the main backbone of the purified bioflocculant with no detection of protein. Energy Dispersive X-ray analysis affirmed the presence of chlorine, phosphorous, oxygen and chlorine as representatives of elemental composition. Thermogravimetric (TGA) analysis revealed over 60% weight was retained at a temperature range of 700 °C. The purified bioflocculant remarkably removed chemical oxygen demand, biological oxygen demand and turbidity in brewery wastewater. This study suggested that the bioflocculant might be an alternate candidate for wastewater treatment.

## Introduction

The continuous discharge of industrial wastewater into water bodies such as rivers and lakes has resulted in water pollution. Due to vast industrialization, the rate of water contamination increases daily, posing a serious threat to terrestrial and aquatic habitats^1^. Thus, the treatment of wastewater and removal of heavy metals before discharging into the water bodies requires rapt attention from researchers and industrial stakeholders^2^. The discharge of poorly or partially treated brewery wastewater into water systems have been associated with many health-related issues. Brewery operations contribute significantly to global environmental problems by consuming water, producing wastewater, producing solid waste, consuming energy, and emitting pollutants into the air. Consequently, these shortcomings have led to environmental problems associated with lack of water, abnormal growth of unwanted organisms responsible for the death of aquatic^3^ and challenges with the health of the communities living around the discharge areas^4^. As a result, there is a need for urgent attention in treating brewery wastewaters before their final discharge into water bodies.

Chemical flocculants are widely utilized in fermentation, downstream processing, and wastewater treatment due to their cheaper purchase cost and higher flocculating efficiency^5,6^. However, its use has been linked to health problems. For example, when aluminium is used as a coagulant in wastewater treatment, the treated effluent may include a higher concentration of aluminium than raw water. Furthermore, research has established that aluminium salts cause Alzheimer's disease, and the usage of polyacrylamide monomers as a flocculant is neurotoxic and carcinogenic^7^. Thus, to eliminate or reduce the risk associated with the use of chemical flocculants, there is a need to develop and explore eco-friendly biodegradable flocculants capable of reducing turbidity in surface water effluents^8,9^. Microorganisms such as bacteria, fungi, yeast and actinomycetes isolated from water, soil and sludge have been reported to produce bioflocculants^10^. It has been established in the literature that water, soil and sludge have served as a good reservoir for bioflocculant producing organisms.

*Bacillus* *velezensis* was first reported in 2005^11^, and several strains have lately been examined for their potential as biopesticides^12,13^. It is non-pathogenic and does not pollute the environment. Its metabolites are widely distributed and display broad-spectrum antibacterial action and strong stress resistance^14^. As a result, there is a tremendous increase in exploring its properties and biotechnological applications. Despite the biopesticide potential of *B.* *velezensis* against a wide range of plant pathogens, it is worthy to note that the strain could flocculate kaolin clay suspension and also serve as an alternative in wastewater treatment. Remarkably, *B.* *velezensis* has been employed in flocculation^15^; however, there is a scarcity of information on the optimal growing conditions for producing the bioflocculant and its industrial application in biotechnology.

Aside from bioprospecting for novel bioflocculant-producing bacteria, options for optimizing microbial product yields include manipulating nutritional conditions. The traditional one-factor-at-a-time technique of medium optimization is rigorous and time-consuming, especially when the number of variables is enormous. Using statistical approaches in microbial systems is a more efficient alternative. Plackett–Burman (PB) design is an efficient and economical technique for screening for crucial factors among a large number of variables with a small number of experiments; it permits the assessment of random error variability and testing of parameter statistical significance^16,17^. Response surface methodology (RSM) is a powerful statistical technique that utilizes quantitative data from relevant experiments to appraise multiple parameters and their interactions, determines the optimal conditions for factors to produce desired responses, and evaluates the relative significance of influencing factors even when complex interactions exist^18^. The techniques have already been successfully utilized to model flocculation processes^19–21^.

Thus, this study describes the modeling, optimization, characterization, and application of bioflocculant produced by *Bacillus* *velezensis*. Several operating variables were screened using the PB design, and the critical process variables were optimized using the central composite design (CCD) of RSM. The biotechnological testing of the bioflocculant was further performed to validate its efficiency in brewery wastewater treatment.

## Materials and methods

### Isolation and cultivation of bioflocculant-producing strain

A sludge wastewater sample was collected from KwaZulu-Natal Province of South Africa, and approximately 1 g of the sample was serially diluted with 9 mL of distilled water (10^1^–10^7^ folds). Afterwards, 1.0 mL of each dilution was plated onto agar plates. The agar plates consist of the following components (g/L): 20 glucose, 5.0 urea, 0.5 yeast extracts, 5.0 K_2_HPO_4_, 2.0 KH_2_PO_4_, 2.0 MgSO_4_, 5 NaCl, and 16 agar. The plates were then incubated for 3–5 days at 30 °C in an incubator. Following cultivation, each colony was further purified on isolation agar plates and kept at 4 °C in the refrigerator. Cultivation of bioflocculant producing strains was performed according to the protocol of Agunbiade, et al.^22^. The composition of production medium includes 1% glucose, 0.1% peptone, 0.03% MgSO4.7H_2_O, 0.5% K_2_HPO_4_, and 0.2% KH_2_PO_4_ in distilled water).

### Assessment of flocculating activity

This was carried out using jar tests by adopting the protocol of Agunbiade, et al.^22^, with modifications. The optical density (OD) of the clarifying solution was determined using a spectrophotometer (UV/Visible Biowave II and Biowave II^+^ England) at 550 nm. The control experiment was conducted in the same manner as the preceding one, except that 2 mL of production medium was used to replace the cell-free supernatant.

Flocculating activity (FA) was evaluated using Eq. (1) below:

1 $$FA{\text{ (\% ) = }}\frac{B - A}{B} \times 100{ }$$

where A is the absorbance of the sample experiment and B is the absorbance of control at 550 nm, respectively.

The strain that exhibited optimum flocculating activity was selected for further study.

### PCR amplification and sequencing of 16SrRNA gene sequences from the pure isolate

DNA extraction of the pure culture was performed using Powersoil® DNA isolation kit (Mo BIO Laboratories, Inc., United States) following the manufacturer’s instructions. Universal bacterial 16S rRNA gene primers 27F (5’-AGA GTT TGA TCC TGG CTC AG-3’) and 1492R (5’-GGT TAC CTT GTT ACG ACT T-3’)^23^ were used to amplify the 16S rRNA gene sequence of the extracted DNA. The PCR standard reaction of a total volume of 25 μL consisted of DNA template (25 ng), 1.5 μL (0.3 μM) of each primer, 12.5 μL KAPA HiFi HotStart Ready-mix (1×) (ROCHE, Randburg, South Africa) and 7 μL nuclease-free water (WhiteSci, Cape Town, South Africa). The PCR program used is as follows: an initial denaturation step of 95 °C for 3 min followed by 25 amplification cycles consisting of 98 °C for 30 s, 65 °C for 30 s and 72 °C for 30 s, followed by a final extension step at 72 °C for 5 min. The PCR product was assessed by gel electrophoresis. Sequencing PCR was performed on the purified PCR product and then purified using the traditional ethanol precipitation method^24^. The purified sequencing product was sequenced to completion at the University of the Free State, Bloemfontein, South Africa. The quality-filtered 1.5-kbp consensus 16S rRNA gene sequence was submitted to the National Center for Bioinformatics Information (NCBI) database (http://blast.ncbi.nlm.nih.gov) to establish the identity.

### Experimental design matrix for bioflocculation activity

#### Plackett–Burman (PB) design for screening

PB is a design tool developed to screen *n* components in *n* + 1 experimental studies^16^. In comparison to the typical full factorial design, the PB design significantly minimizes the trial numbers required to achieve the desired outcome, lowering the resource cost in terms of labour and time. The present study explored 8 independent medium variables using a PB design to determine which variables significantly affected bioflocculation activity. These components were: glucose, MgSO_4_^.^7H_2_O, NaCl, yeast extract, K_2_HPO_4_, (NH_4_)_2_SO_4_, KH_2_PO_4_, and urea. To evaluate each medium variable, two concentration levels + 1 (high) and –1 (low) were chosen. The PB experimental matrix was designed and developed using NCSS version 12 (NCSS, LLC., USA) statistical software following the first-order regression equation provided in Eq. (2):

2 $$Y = b_{0} + \sum\limits_{i = 1}^{k} {b_{i} x_{i} }$$

where the bioflocculation activity is represented as *Y* (the response), the model intercept is *b*_0_, the linear coefficient is *b*_*i*_, the level of the independent variable is represented as *x*_*i*_, and *k* is the number of variables. Although the model was not intended to explain the relationship between variables, it can be used to screen for variables that have a substantial effect on the response^19^. In this study, 8 variables of the medium were chosen and assessed in 12 experimental conditions. Regression analysis was used to identify the variables of great significance on bioflocculation activity (i.e. at 95% level with *p* < 0.05), and these variables were then subjected to additional optimization studies.

#### Central composite design (CCD) for optimization

RSM was used in combination with CCD to further examine the impact of the most critical process variables revealed by the PB design. These independent variables and their chosen ranges were glucose (12–16 g/L), MgSO_4_.7H_2_O (2.5–3.5 g/L), and (NH_4_)_2_SO_4_ (1.2–1.6 g/L) with the bioflocculation activity as the dependent variable (response). Applying fractional factorial design of CCD consisting of five levels and three factors, a total of 20 experimental conditions were generated. The design matrix comprised six center points and six axial points, with an axial distance (α) of ± 1.68 to transform the design to its orthogonal form. The bioflocculation activity was fitted to a second-order regression model to establish a link between the dependent and independent variables using Eq. (3):

3 $$Y = \delta_{0} + \delta_{1} A + \delta_{2} B + \delta_{3} C + \delta_{12} AB + \delta_{13} AC + \delta_{23} BC + \delta_{11} A^{2} + \delta_{22} B^{2} + \delta_{33} C^{2}$$

where *Y* is the bioflocculation activity (response), *δ*_0_ is the intercept term; *δ*_1_, *δ*_2_, and *δ*_3_ are the coefficients of the linear terms; *δ*_12_, *δ*_13_, and *δ*_23_ are the coefficients of the interaction terms; *δ*_11_, *δ*_22_, and *δ*_33_ are the coefficients of the quadratic terms.

Each experimental condition was repeated twice, and the mean bioflocculation activity for each response was determined. The experimental matrix was created using Design-Expert version 12.0 (Stat-Ease Inc., USA) software and was also used for statistical analysis of the data.

#### Purification of the bioflocculant

The strain SW2 bioflocculant was purified according to the modified method adopted by Bar-Or and Shilo^25^. The isolate was cultivated in an optimal fermentation medium that resulted in the highest flocculating activity when tested against kaolin clay suspension.

#### Characterization of purified bioflocculant

The total protein content and total sugar content were determined in this study. Lowry's procedure was used to evaluate the total protein content of purified bioflocculant using Bovine Serum Albumin as a standard^26^. The total sugar content of the bioflocculant was determined using the phenol–sulfuric technique^27^, and glucose was used as the standard solution. The functional groups of the purified bioflocculant were examined using a Fourier transform infrared (FTIR) spectrophotometer (Perkin Elmer System 2000, England). A thermal analyzer (STA 449/C Jupiter, Netzsch, Germany) was used for thermogravimetric analysis (TGA) at a heating rate of 10 °C/min and a steady flow of nitrogen gas across a temperature range of 20 to 900 °C. The surface morphology of the sample was captured using a Tescan MIRA3 RISE Scanning Electron Microscope (SEM), and the elemental composition was determined using a Thermo Fisher (Nova NanoSEM) coupled to an energy-dispersive X-ray (EDX) detector.

#### Dosage concentration and wastewater flocculation studies

Brewery wastewater was collected from South African Brewery, Gauteng Province of South Africa and the sample was transported to the laboratory for processing. The efficiency of the purified bioflocculant was validated by treating the wastewater with purified bioflocculant prepared at different concentrations. Briefly, each dosage concentration (0.1–1.0 mg/mL) and 3 mL of 1% CaCl_2_ was added to 100 mL of the brewery wastewater. Coagulation/flocculation was achieved by continuously stirring the suspension by a six-paddle stirrer at 160 rpm for 2 min and then at 40 rpm for 2 min. Afterwards, brewery wastewater was allowed to sediment for 5 min, and 2 mL of the clarifying upper layer was gently withdrawn for further analysis. The residual chemical oxygen demand (COD) and turbidity were measured using a spectrophotometer (DR 3800) and turbidimeter (HACH, USA). The removal efficiency (RE) was calculated as shown in Eq. (4):

4 $$RE = \frac{{C_{0} - C}}{{C_{0} }} \times 100{ }$$

where *C*_*o*_ is the initial value and *C* is the value after the flocculation treatment.

The biological oxygen demand (BOD) was calculated by adding 25 mL of untreated and 50 mL of treated brewery wastewater samples in BOD bottles that were then filled with BOD buffer; this served as the working solution and incubated for 5 days at 20 °C. The initial and final dissolved oxygen (DO) concentrations were measured using a HI5421 BOD Meter (Hanna, USA) after 15 min and 5 days, respectively. To calculate the BOD and the percentage BOD removal efficiency, Eq. (5) was used.

5 $$BOD = \frac{{D_{1} - D_{2} }}{P}{ }$$

where D_1_ is the DO in the diluted specimen after preparation, D_2_ is the DO after 5 days, and P is the decimal fraction of the specimen used.

6 $$RE(\% ) = \frac{{B_{1} - B_{2} }}{{B_{1} }}{ } \times {100}$$

where *B*_*1*_ is the untreated sample and *B*_*2*_ is the treated sample.

### Statistical investigation

Using SPSS 16.0, the results were reported as means standard deviation of three replicates and subjected to one-way analysis of variance (ANOVA) followed by Duncan multiple range tests to find significant differences in all parameters. At *p* < 0.05, values were considered statistically significant.

## Results and discussion

### Isolation and identification of bioflocculant producing organism

Twenty-six strains isolated from wastewater sludge were screened for their potential in flocculating kaolin clay suspension. The initial screening performed affirmed that strains SW1, SW2, and SW3 exhibited flocculating activity exceeding 60%. The 16S rRNA gene sequencing of the confirmed strains is shown in Table 1. It is worthy to note that SW2 exhibited flocculating activity of over 89% against kaolin clay suspension. Hence, SW2 was selected for further investigation. Basic Local Alignment Search Tool (BLAST) analysis of the nucleotide sequence of the 16S rRNA gene affirmed the bacterium (SW2) has 100% similarity to *Bacillus* *velezensis*, and the sequence was deposited in Genbank as *Bacillus* *velezensis* with accession number MN714634.

**Table 1:** 16S rRNA gene sequences based on BLAST analysis.

Sample	(Accession no)	Related organism	Phylogenetic group	% similarity
SW1	(MN714633)	*Stenotrophomonas* *maltophilia*	Gammaproteobacteria	100
SW2	(MN714634)	*Bacillus* *velezensis*	Firmicutes	100
SW3	(MN714635)	*Bacillus* *subtilis*	Firmicutes	100

### Variables influencing the activity of bioflocculation

The design pattern generated by the PB design and the corresponding bioflocculation activity from each experimental condition is depicted in Table 2. Regression analysis results of the screened variables are illustrated in Table 3.

**Table 2:** Experimental and corresponding results by Plackett–Burman (PB) design for 8 variables.

Runs	Variables and their levels								Bioflocculation activity (%)	
	*x*_1_	*x*_2_	*x*_3_	*x*_4_	*x*_5_	*x*_6_	*x*_7_	*x*_8_	Experimental	Predicted
1	1 (12.5)	1 (2.5)	–1 (0.6)	1 (25)	1 (6.25)	1 (2.5)	–1 (1)	–1 (0.5)	22.19	18.46
2	1 (12.5)	–1 (2)	1 (0.75)	1 (25)	1 (6.25)	–1 (2)	–1 (1)	–1 (0.5)	33.91	37.64
3	–1 (10)	1 (2.5)	1 (0.75)	1 (25)	–1 (5)	–1 (2)	–1 (1)	1 (0.7)	35.16	38.44
4	1 (12.5)	1 (2.5)	1 (0.75)	–1 (20)	–1 (5)	–1 (2)	1 (2)	–1 (0.5)	40.25	32.57
5	1 (12.5)	1 (2.5)	–1 (0.6)	–1 (20)	–1 (5)	1 (2.5)	–1 (1)	1 (0.7)	13.14	16.87
6	1 (12.5)	–1 (2)	–1 (0.6)	–1 (20)	1 (6.25)	–1 (2)	1 (2)	1 (0.7)	81.86	85.14
7	–1 (10)	–1 (2)	–1 (0.6)	1 (25)	–1 (5)	1 (2.5)	1 (2)	–1 (0.5)	77.27	76.60
8	–1 (10)	–1 (2)	1 (0.75)	–1 (20)	1 (6.25)	1 (2.5)	–1 (1)	1 (0.7)	71.53	63.85
9	–1 (10)	1 (2.5)	–1 (0.6)	1 (25)	1 (6.25)	–1 (2)	1 (2)	1 (0.7)	90.80	87.52
10	1 (12.5)	–1 (2)	1 (0.75)	1 (25)	–1 (5)	1 (2.5)	1 (2)	1 (0.7)	72.22	72.89
11	–1 (10)	1 (2.5)	1 (0.75)	–1 (20)	1 (6.25)	1 (2.5)	1 (2)	–1 (0.5)	52.67	60.35
12	–1 (10)	–1 (2)	–1 (0.6)	–1 (20)	–1 (5)	–1 (2)	–1 (1)	–1 (0.5)	39.10	39.77

**Table 3:** Plackett–Burman design for the screening of variables at selected concentrations and the regression analysis.

No	Variables	Concentration (g/L)		Estimate	*t* value	*p* value
		–1 level	+ 1 level			
*x*_1_	Glucose	10	12.5	–0.3518	–3.239	0.0479^a^
*x*_2_	MgSO_4_.7H_2_O	2	2.5	–0.4158	–3.828	0.0314^a^
*x*_3_	Yeast extract	0.6	0.75	–0.0636	–0.586	0.5991
*x*_4_	NaCl	20	25	0.1128	1.038	0.3755
*x*_5_	K_2_HPO_4_.3H_2_O	5	6.25	0.2591	2.386	0.0971
*x*_6_	KH_2_PO_4_	2	2.5	–0.0412	–0.379	0.7296
*x*_7_	(NH_4_)_2_SO_4_	1	2	0.6835	6.294	0.0081^a^
*x*_8_	Urea	0.5	0.7	0.3394	3.125	0.0523

^a^Statistically significant at the confidence level of 95%.

Following the results, a first-order regression was established to explain the bioflocculation activity, as shown in Eq. (7).

7 $$Bioflocculation \, activity \, \left( \% \right) \, = { 62}.{7133 }{-}{ 6}.{864}x_{{1}} {-}{ 4}0.{56}x_{2} {-}{ 2}0.{6889}x_{3} + { 1}.{1}x_{4} + { 1}0.{1}0{93}x_{5} {-}{ 4}.0{2}x_{6} + { 33}.{34}x_{7} + { 92}.{7667}x_{8}$$

As presented in Table 3, the regression analysis for the screened variables showed that glucose (*x*_1_), MgSO_4_^.^7H_2_O (*x*_2_), and (NH_4_)_2_SO_4_ (*x*_7_) had a positive influence on bioflocculation activity, while yeast extract (*x*_3_), NaCl (*x*_4_), K_2_HPO_4_.3H_2_O (*x*_5_), KH_2_PO_4_ (*x*_6_), and urea (*x*_8_) contributed negatively.

The eminent influence of (NH_4_)_2_SO_4_, MgSO_4_.7H_2_O, and glucose on the bioflocculation activity was due to their necessities as constituents for considerable cell growth. In synthesizing polysaccharides from a *Porphyridium* sp.^28^, ammonium appeared to be an important component and was also reported as an essential nitrogen source on bioflocculant synthesis using a mixed culture of *Streptomyces* and *Cellulomonas* species. Glucose has also been reported as an important carbon source in the evaluation of bioflocculant yield^19,20^. Earlier research established that ratios of C/N were critical in microbe metabolic processes, such as modifying the composition of fatty acids produced by the heterotrophic *Chlorella* *sorokiniana* and strengthening biological hydrogen production by *Clostridium* *pasteurianum*^29^. Additionally, magnesium has been identified as a critical inorganic ion that influences various physiological processes, including enzyme activity, cell development, and cell division^19,30^. Hence, it was expected that the presence of magnesium was crucial in the metabolic pathway of *B.* *velezensis* that would be ineffectual if substituted by another cation.

### Modeling results of bioflocculation process

The results of the bioflocculation process carried out with *B.* *velezensis* are presented in Table 4.

**Table 4:** Central composite design (CCD) matrix with the experimental and predicted bioflocculation activities.

Run no	Space type	Glucose (g/L)	MgSO_4_.7H_2_O (g/L)	(NH_4_)_2_SO_4_ (g/L)	Bioflocculation activity (%)	
		*A*	*B*	*C*	Experimental	Predicted
1	Axial	10.6364	3	1.4	93.0	93.36
2	Factorial	16	2.5	1.2	94.0	94.53
3	Factorial	12	3.5	1.6	96.3	95.76
4	Axial	14	3.8409	1.4	95.3	95.50
5	Center	14	3	1.4	95.6	95.67
6	Center	14	3	1.4	95.3	95.67
7	Factorial	16	3.5	1.6	96.8	96.88
8	Factorial	16	3.5	1.2	95.1	95.14
9	Center	14	3	1.4	96.5	95.67
10	Factorial	12	2.5	1.2	92.3	92.21
11	Axial	14	3	1.73636	96.7	97.04
12	Factorial	12	2.5	1.6	93.7	93.65
13	Center	14	3	1.4	95.7	95.67
14	Center	14	3	1.4	95.2	95.67
15	Axial	14	2.1591	1.4	93.4	93.21
16	Factorial	16	2.5	1.6	96.6	96.52
17	Factorial	12	3.5	1.2	94.5	94.57
18	Axial	14	3	1.06364	94.7	94.37
19	Axial	17.3636	3	1.4	96.6	96.25
20	Center	14	3	1.4	95.7	95.67

The experimental bioflocculation activity ranged from 92.3 to 96.8%. By the results obtained, a second-order model equation was developed in terms of the coded factors expressing the bioflocculation activity (response) to the independent variables. This is described in Eq. (8):

8 $$Bioflocculation \, activity \, \left( \% \right) \, = { 95}.{67 } + \, 0.{86}0{7}A + \, 0.{68}0{6}B + \, 0.{\text{7955C }}{-} \, 0.{4375}AB + \, 0.{1375}AC{-} \, 0.0{625}BC{-} \, 0.{3}0{52}A^{{2}} {-} \, 0.{4643}B^{{2}} + \, 0.0{13}0C^{{2}}$$

where, *A* is glucose concentration (g/L), *B* is MgSO_4_.7H_2_O concentration (g/L), and *C* is (NH_4_)_2_SO_4_ concentration (g/L).

The significance and appropriateness of the model developed were statistically evaluated using ANOVA. Diagnostic plots were used to evaluate the influence of each model term and their interaction with bioflocculation activity. Table 5 summarizes the ANOVA results and regression analyses performed on the developed model. The *F*-value and *p*-value of the model obtained as 15.59 and < 0.0001, respectively, signal the statistical significance at a 95% confidence level^19,20^. The significance of the process variables was appraised using the *p*-value. The *p*-value represents the probability error which is employed to check if the individual regression coefficient is significant or not^31^. A term with a *p*-value < 0.05 shows its significance in the model. Observation from Table 5 showed that all the linear terms (i.e. glucose, MgSO_4_⋅7H_2_O, and (NH_4_)_2_SO_4_) of the model are significant. The interaction between glucose and MgSO_4_⋅7H_2_O is significant, while other interaction terms within the model are not significant. Apart from the quadratic term of (NH_4_)_2_SO_4_, all other quadratic terms tested are significant. The lack-of-fit value of 0.4611 obtained for the developed model was *not* *significant*, which further demonstrated the validity of the model to predict the bioflocculation activity within the selected ranges.

**Table 5:** Analysis of variance (ANOVA) results for the regression model of *B.* *velezensis* bioflocculation activity.

Source of variance	Sum of squares	DF	Mean square	*F* value	*p* value		Quality of fit and statistical indices	
							Parameter	Value
Model	30.98	9	3.44	15.59	< 0.0001^a^	Significant	*R*^2^	0.9335
*A-*Glucose	10.12	1	10.12	45.82	< 0.0001^a^		Adjusted *R*^2^	0.8736
*B*-MgSO_4_⋅7H_2_O	6.33	1	6.33	28.66	0.0003^a^		Predicted *R*^2^	0.6800
*C*- (NH_4_)_2_SO_4_	8.64	1	8.64	39.14	< 0.0001^a^		Signal-to-noise ratio	14.5380
*AB*	1.53	1	1.53	6.94	0.0250^a^		Standard deviation	0.4699
*AC*	0.1512	1	0.1512	0.6850	0.4272		Mean	95.15
*BC*	0.0313	1	0.0313	0.1415	0.7146		% CV	0.4938
*A*^2^	1.34	1	1.34	6.08	0.0334^a^			
*B*^2^	3.11	1	3.11	14.07	0.0038^a^			
*C*^2^	0.0025	1	0.0025	0.0111	0.9181			
Residual	2.21	10	0.2208					
Lack of fit	1.15	5	0.2309	1.10	0.4611	Not significant		
Pure error	1.05	5	0.2107					
Corrected total SS	33.19	19						

*DF* degree of freedom, *CV* coefficient of variation.

^a^Statistically significant at the confidence level of 95%.

The reliability of the regression model was inspected via other statistical indicators, namely; coefficient of determination (R^2^), predicted R^2^, adjusted R^2^, signal-to-noise ratio, and % coefficient of variance (CV). The obtained R^2^ value of 0.9335 indicates that the model can account for 93.35% variation in the bioflocculation activity, thus confirming a satisfactory connection between the experimental and predicted bioflocculation activities^19,32^. This precision was sustained by the adjusted R^2^ value of 0.8736 and the predicted R^2^ value of 0.6800 estimated for the model since the difference between the two statistical parameters did not exceed the allowable standard of 0.2^33^. The signal-to-noise ratio of 14.54 estimated for the model was greater than the minimum value of 4 required for a good model^31^. The high value evaluated shows a good signal in the model to coordinate the design space for the experimental outcomes. Furthermore, the low CV value of 0.49% indicates that the experimental and predicted data by the model correlate well. CV value of < 10% is always required^31,32^.

**Figure 1:**
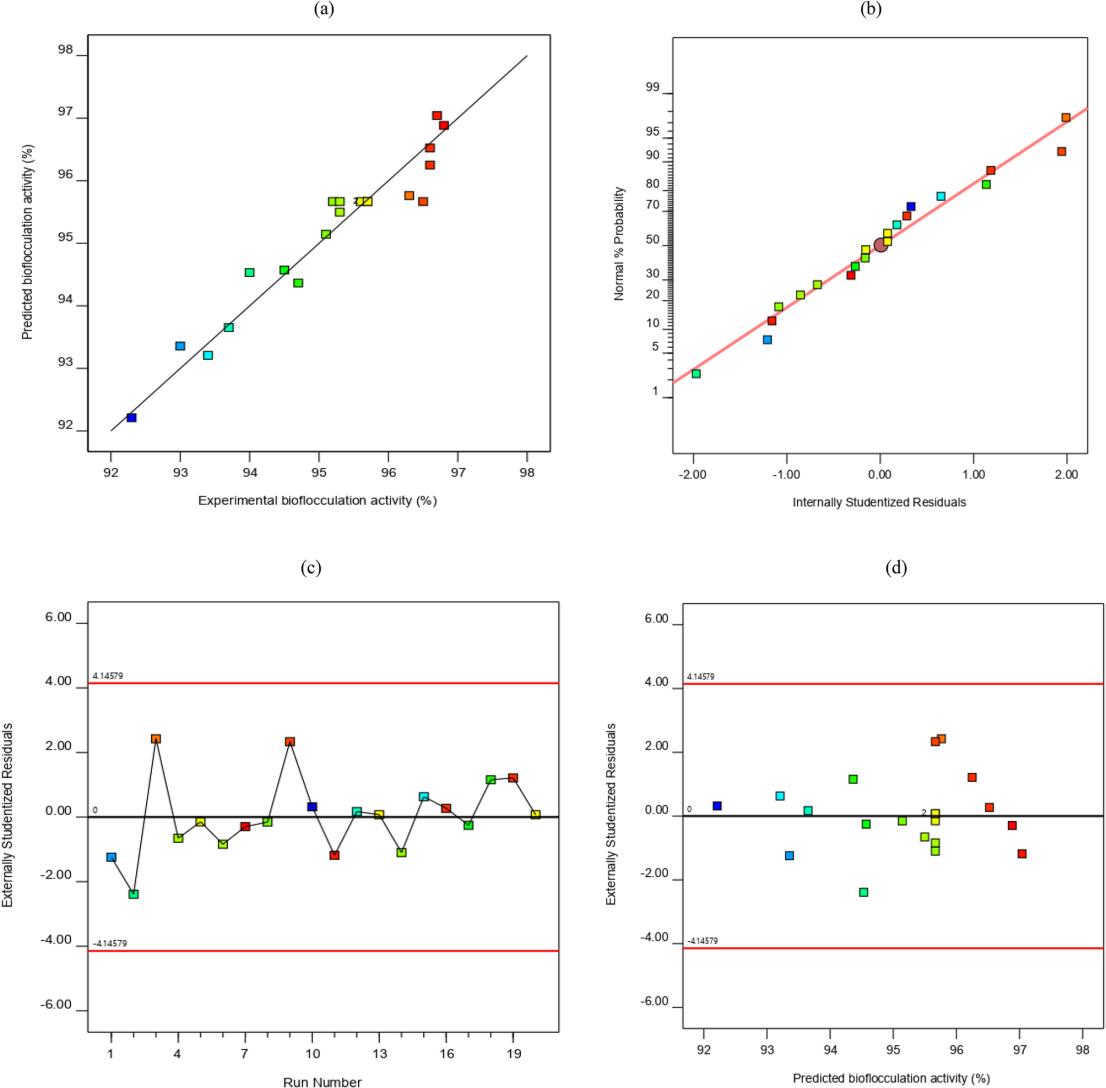
Model adequacy plots of bioflocculation activity (**a**) predicted response *vs* experimental response; (**b**) normal plot of residuals; (**c**) residuals *vs* predicted, and (**d**) outlier *t* plot.

It is not sufficient to rely on the ANOVA generated results to interpret the adequacy of a developed model. Therefore, the reliability of the model was further explored via diagnostic plots (Fig. 1). The plot of experimental data against the predicted data of bioflocculation activities is presented in Fig. 1a. The alignment of data points with the 45° line demonstrates an acceptable concurrence between the experimental and predicted data, hence revealing a great assessment of the dependent variable to changes in the process variables (i.e. glucose, MgSO_4_.7H_2_O, and (NH_4_)_2_SO_4_). The dependability of the regression equation should be verified to know whether the residuals follow a normal distribution^34^. The difference between the experimental and predicted data for each experimental condition is termed residual. It indicates the extent to which a regression model obeys the ANOVA assumption^31,32^. The normal distribution was plotted against studentized residuals, as shown in Fig. 1b. The plot followed a normal distribution since most data points aligned along the straight line^34^. This signals that the residuals followed a normal distribution. Studentized residuals were plotted against predicted bioflocculation activity, as shown in Fig. 1c. It was observed that the residuals are randomly scattered, showing the applicability of the model that the real observed data is unrelated to the response data^31,32^. Figure 1d illustrates the outlier *t* plot for each experimental run during the bioflocculation process. Residual with a huge value can be identified by looking at the plot. This plot explains the degree to which experimental data digress from the predicted data. As can be observed, all residuals fall inside the 95% confidence interval (± 3.00); this signals no problem with the developed model and indicates that transformation is not needed for the response variable^31,32^.

### Interactive influence of process variables on bioflocculation activity

**Figure 2:**
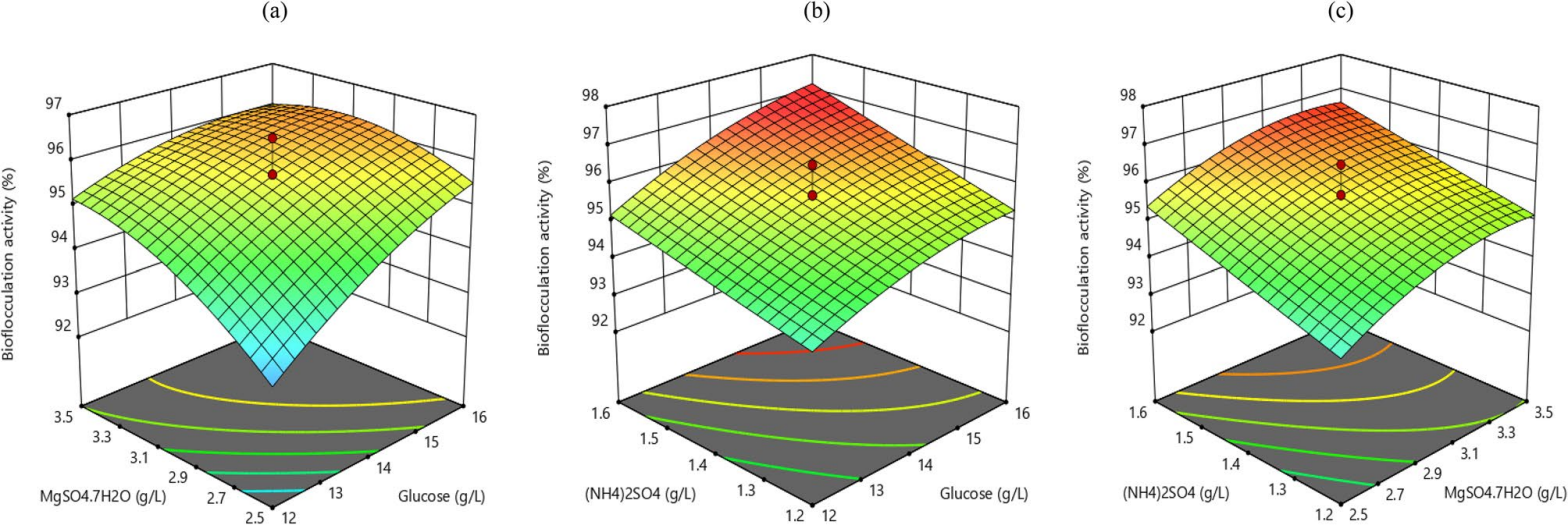
Surface plots of the influence of pertinent variables on the bioflocculation activity.

Response surface plots were used to assess the influence of the three independent variables (glucose, MgSO_4_.7H_2_O, and (NH_4_)_2_SO_4_) on the bioflocculation activity. Figure 2a displays the influence of MgSO_4_⋅7H_2_O and glucose. The increase in both variables led to an increase in the bioflocculation activity till it attains a maximum value of 96.8%. Figure 2b shows the influence of (NH_4_)_2_SO_4_ and glucose. From the plot, as both variables increase, bioflocculation activity increases as well, until a maximum value was attained. Figure 2c displays the interactive influence between (NH_4_)_2_SO_4_ and MgSO_4_⋅7H_2_O. As visualized from the plot, the increase in both variables also led to an increase in the bioflocculation activity till it attains a maximum. Thus, the results obtained showed that the ranges selected for the variables were sufficient in producing high bioflocculation activity. The observed interaction between the independent variables and the response reported in this study corroborated well with the studies conducted by He, et al.^19^ and Nwodo, et al.^20^, where flocculation yield was also the objective of the study.

### Model validation of optimal condition

The in-built optimization tool of RSM was applied to search for the optimal condition of the significant input variables to maximize the bioflocculation activity. This was done by setting the process input variables constraint within the ranges investigated, followed by setting the goal of bioflocculation activity to the maximum. The optimal condition predicted by the software for the bioflocculation process with the desirability function of 1 is glucose concentration of 15.91 g/L, MgSO_4_⋅7H_2_O concentration of 3.05 g/L, and (NH_4_)_2_SO_4_ concentration of 1.59 g/L with corresponding optimum bioflocculation activity of 97.11%. The optimal condition was used to perform experiments in duplicate, and the bioflocculation activity of 97.04 ± 0.03% was obtained. The closeness of this value with the predicted value signifies the efficiency of the regression model developed to describe the bioflocculation process. The optimization result obtained in this present study agreed well with an earlier study that used a consortium of *Streptomyces* sp. Gansen and *Cellulomonas* sp. Okoh, where optimum bioflocculant production of 98.9% was obtained under sucrose concentration of 15 g/L, peptone concentration of 1.5 g/L, and MgCl_2_ concentration of 1.6 g/L^20^.

### Characterization of bioflocculant produced by *B. velezensis*

**Figure 3:**
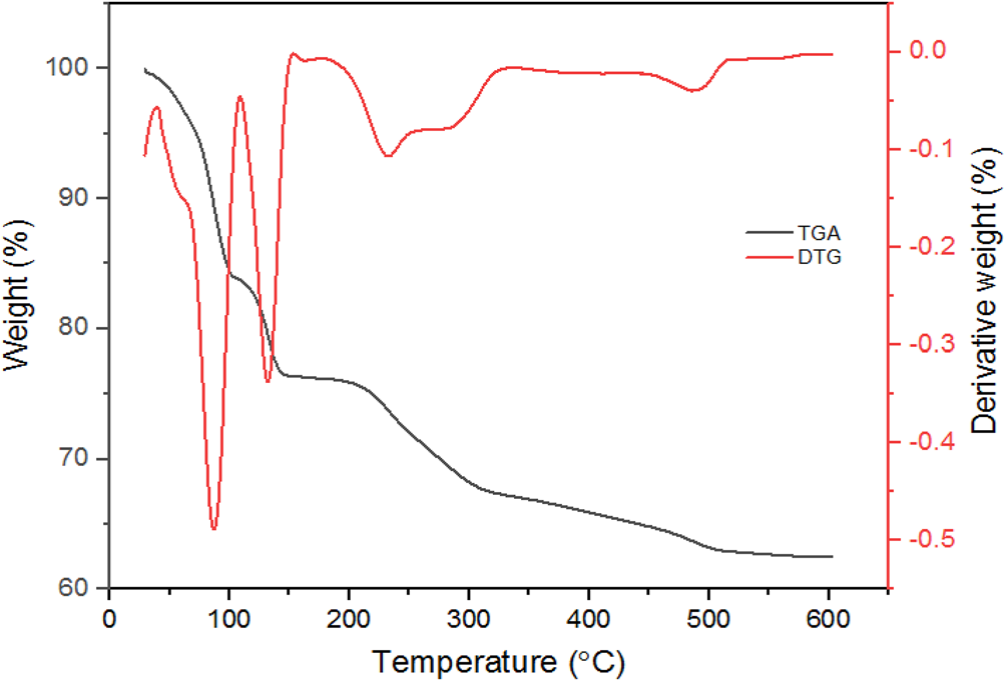
TGA/DTA analysis of purified bioflocculant.

Chemical analysis showed the presence of 83% total polysaccharide content with no protein detection in the bioflocculant structure, thus, reaffirming the stability of the bioflocculant at higher heating temperatures. Furthermore**,** Fig. 3 revealed the weight loss phases resulting from the thermal decomposition of the purified bioflocculant. The loss of around 15.80% of the initial weight of the material at around 87 °C is attributed to the loss of physically adsorbed moisture within the matrix of the sample. The moisture could be responsible for the presence of a carboxyl group in the bioflocculant, which is responsible for the interaction with water molecules^35^. The decomposition observed at 132, and 233 °C could be the organic carbon left in the material. After heating the material at ~ 491 °C, nearly 60% of the weight was maintained, which confirmed the stability of the bioflocculant produced by the test organism.

**Figure 4:**
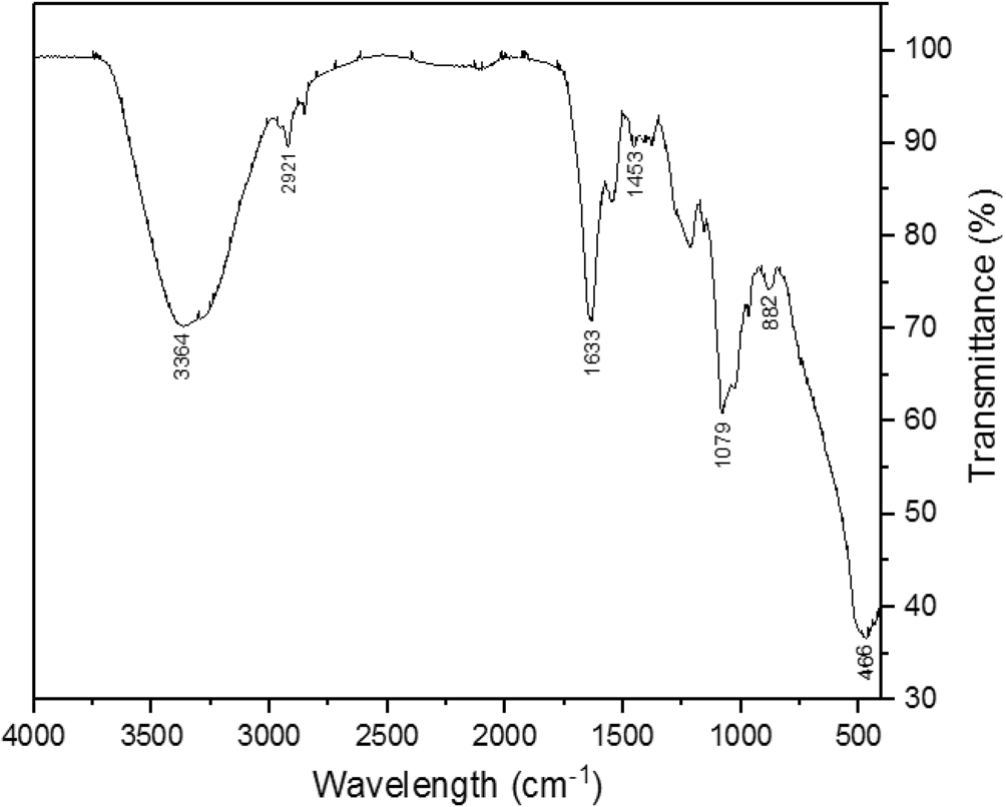
FTIR spectrum of purified bioflocculant.

The FTIR spectrum of the purified bioflocculant is displayed in Fig. 4. The intense absorption peak at 3364 cm^–1^ could be attributed to the vibration of the hydroxyl group in the sugar ring of the polysaccharides. A weak band at 2921 cm^–1^ is representative of C-H aliphatic bands^36^. The band at 1633 and 1453 cm^–1^ could be due to carboxylate ions, suggesting uronate in the polysaccharide^15^. Furthermore, the peak at 1079 cm^–1^ could be attributed to methoxyl groups, and the peak at 882 cm^–1^ may indicate β-configuration of the main glucan linkages^37^. The presence of the hydroxyl group enhances the site at which the bioflocculant dissolves in water. On the other hand, carboxylate and methoxyl groups serve as the binding sites for the Ca^2+^ divalent cation, increasing the floc formation rate. As a result, the interaction of these groups with divalent cations allows bioflocculant to adsorb onto the surface of the particles, enhancing aggregation and facilitating kaolin clay suspension to settle faster. Thus, confirming the polar functional groups in the bioflocculant to be responsible for flocculation. This observation is consistent with the report of Yang et al.^38^.

**Figure 5:**
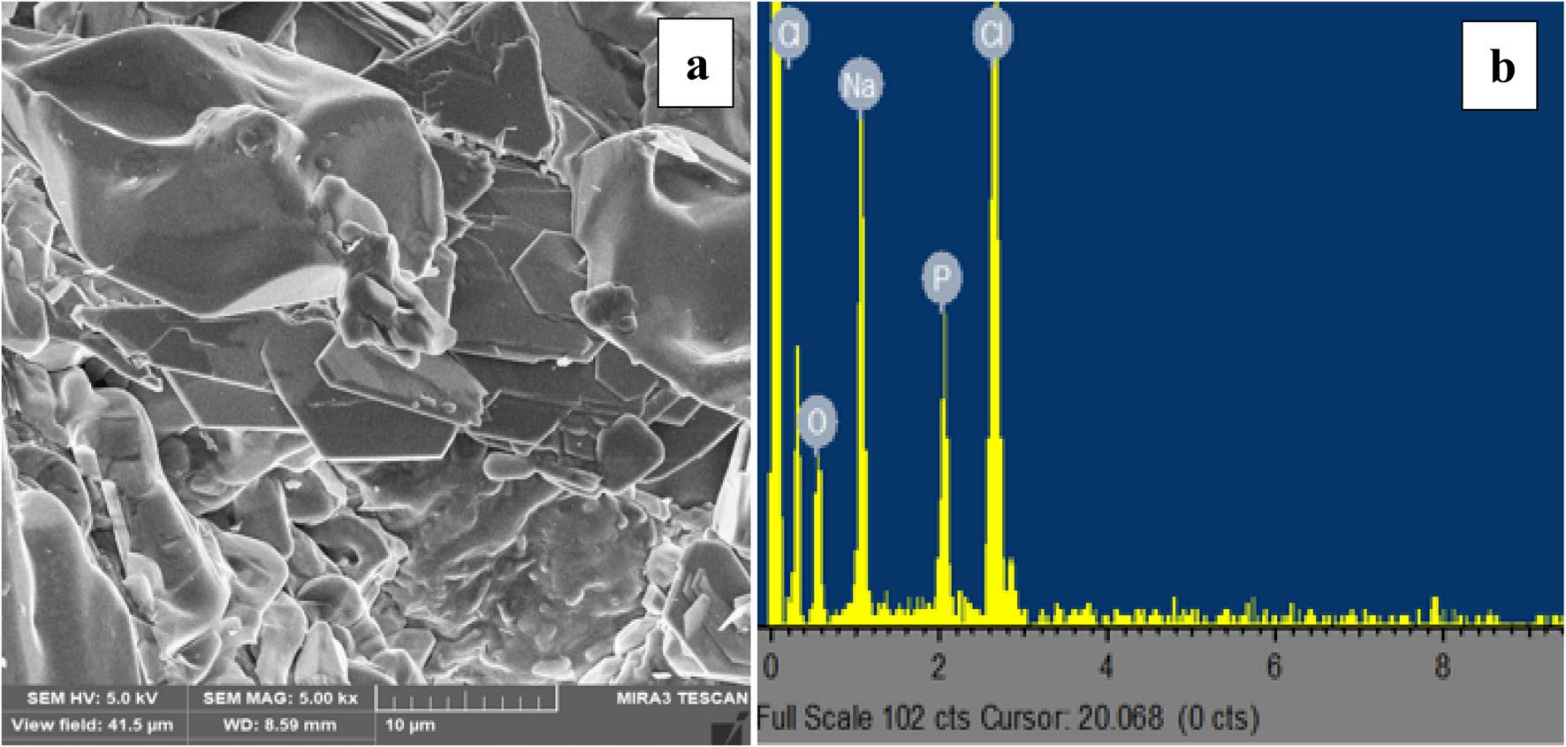
(**a**) SEM image and (**b**) EDX analysis of purified bioflocculant.

The surface morphology structure of the purified bioflocculant is shown in Fig. 5a. The image revealed the bioflocculant produced by *B.* *velezensis* has a rough, compact, and irregular pattern. This configuration in the shape of the bioflocculant may be responsible for its efficient flocculation. As a result of the interaction between both the bioflocculant and the kaolin clay particle, flocs formed that eventually aggregated to produce bigger sized flocs. Interestingly, the floc precipitated out of the suspension as the result of gravity, thus, affirming that bridging played an important role in the flocculation. This observation corroborates the documented reports on bioflocculants produced by *Arthrobacter* *humicola* *and* *Terrabacter* sp.^10,22^*.* The EDX analysis affirmed the presence of chlorine (28.2%), phosphorous (12.3%), sodium (22.36%), and oxygen (37.14%) (Fig. 5b). Similar elements were also observed in the work of Singh, et al.^39^ on the exopolysaccharide produced by *Bacillus* *licheniformis* and in the work of Okaiyeto, et al.^40^ on the characterization of a bioflocculant (MBF-UFH) produced by *Bacillus* sp AEMREG7.

### Dosage concentration and treatment of brewery wastewater

**Figure 6:**
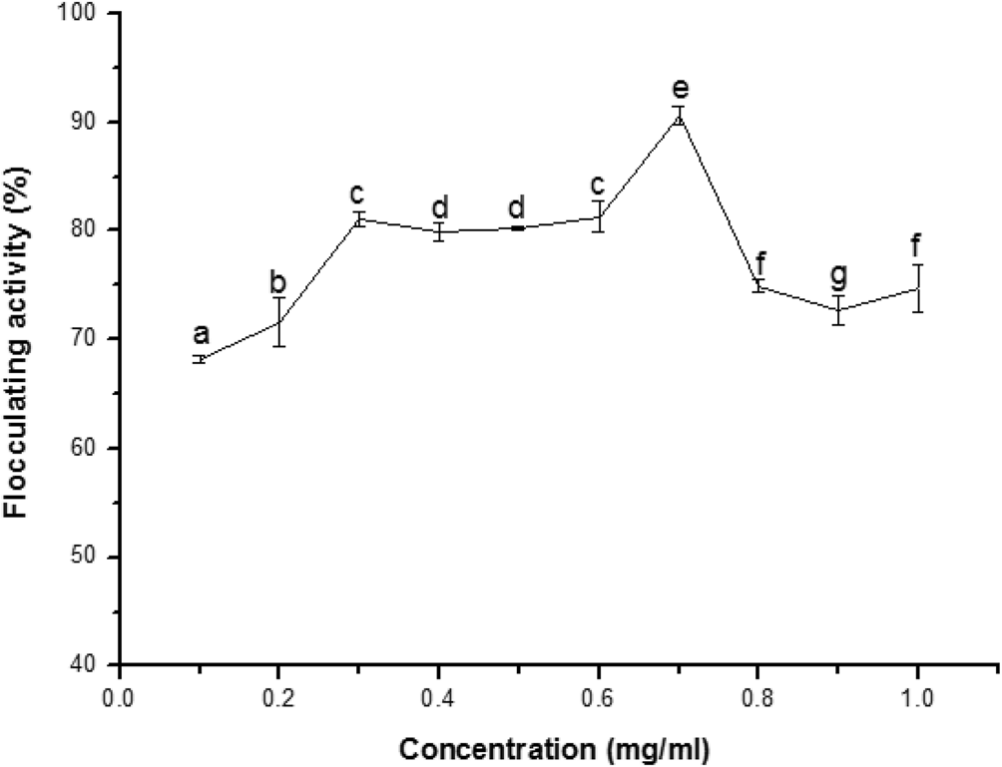
The effect of concentration on the treatment of brewery wastewater. The percentage of flocculating activities of different alphabetic letters differs significantly (p < 0.05).

Choosing the right dosage concentration in the coagulation-flocculation process is paramount in wastewater treatment. As shown in Fig. 6, the optimal dosage requirement for the flocculation of brewery wastewater was achieved at a concentration of 0.7 mg/mL with the optimum flocculating efficiency of > 90%. It is worthy to note that there was no substantial difference in flocculating activity when the concentration was adjusted from 0.3 to 0.6 mg/mL. Conversely, when the dosage was increased from 0.8 to 1.0 mg/mL, there was a sharp decline in bioflocculation efficiency. It has been well reported that low or high dosage might lead to incomplete and/or poor coagulation performance during the process of flocculation^41^. The decrease in flocculating activity at dosage concentrations ranging from 0.8 to 1.0 mg/mL could be attributed to "flocculation deterioration" in which some colloidal particles were obstructed by higher concentrations of flocculant, resulting in a colloid protection function and a decrease in flocculating activity. Hence, validating the correct dosage will minimize the cost of treatment and reduce the risk of health-associated problems.

Brewery wastewater has a high chemical oxygen demand (COD), ranging between 2000 and 6000 mg/L, due to the presence of organic compounds as by-products of the brewing process^42^. The high consumption of beer has led to a larger volume of effluents produced and has necessitated the treatment of the brewery wastewater before its discharge into the environment. From Fig. 6, different concentrations of purified bioflocculants (0.1–1.0 mg/mL) could flocculate brewery wastewater using the Jar test. Optimum flocculating efficiency was evident at a dosage concentration of 0.7 mg/mL. It was observed in this study (Table 6) that the bioflocculant produced by *Bacillus* *velezensis* was successful in removing turbidity, COD, and BOD at efficiencies of 72.0, 62.0, and 53.6%, respectively. Coagulants and flocculants used in wastewater treatment may destabilize particulate matter, resulting in floc formation and improved sedimentation^43^. In another study, bioflocculant was used to treat distillery effluent that resulted in removing 62, 55, 76, and 74% of TDS, TSS, BOD, and COD, respectively^44^. Furthermore, purified bioflocculant was utilized in treating drinking water to minimize turbidity and COD levels^45^. The potential of the purified bioflocculant in COD, BOD, and turbidity removal suggested its practical application in biotechnology and wastewater treatment.

**Table 6:** Application of bioflocculant produced by *B.* *velezensis* in brewery wastewater treatment.

pH		Turbidity		COD (mg/L)		BOD (mg/L)	
A	B	A	B	A	B	A	B
6.28 ± 1.6	6.01 ± 0.8	442 ± 1.8	124 ± 2.6	4033 ± 2.4	1534 ± 1.5	3.365 ± 0.1	1.561 ± 0.6

The values are expressed as means ± standard deviation of triplicate determinations. A: before treatment, B: after treatment.

## Conclusions

This study optimized medium variables for bioflocculant by *Bacillus* *velezensis* using RSM. The PB design was used in the first step to screen and determine the significant variables affecting bioflocculation activity. Screened media components revealed glucose, MgSO_4_.7H_2_O, and (NH_4_)_2_SO_4_ as preferred carbon, cation, and nitrogen source, respectively. CCD combined with RSM was used in the second step to identify the optimal concentration of each significant variable. Maximum bioflocculation activity of 97.11% was predicted for the mathematical regression model at the optimum operating parameters of glucose concentration of 15.91 g/L, MgSO_4_.7H_2_O concentration of 3.05 g/L, and (NH_4_)_2_SO_4_ concentration of 1.59 g/L. This was confirmed in the laboratory as 97.04 ± 0.03%. Characterization of the produced bioflocculant showed the major presence of Na, Cl, P, and O, while the presence of carboxyl and hydroxyl groups showed an enhanced site for bioflocculation activity. The bioflocculant produced proved effective in wastewater treatment with removal efficiencies of 72.0% turbidity, 62.0% COD, and 53.6% BOD. Nonetheless, the discovery of the significance of the carbon to nitrogen ratio and other nutritional sources in optimizing bioflocculation activity is novel. Thus, this study reveals that optimizing the culture medium and growth conditions could lower the cost of medium components while increasing the economic viability of the bioflocculant (SW2) for wastewater treatment and other biotechnological applications.

## Data Availability

The information of DNA sequences analyzed during the current study is available in the GenBank repository, under the accession numbers MN714633, MN714634 and MN714635.
